# A perspective: *PLA2G4A* as drug target for vascular inflammation in Alzheimer's disease

**DOI:** 10.1002/alz.71320

**Published:** 2026-04-08

**Authors:** Ambreen Kanwal, Bilal E. Kerman, Shaowei Wang, Kevin Camey, Boyang Li, Lisi Flores‐Aguilar, Nada Ali, Laura Beth McIntire, Chang April Shu, Stan G. Louie, Elizabeth Head, Zoe Arvanitakis, Hussein N. Yassine

**Affiliations:** ^1^ Department of Genetics Washington University in Saint Louis Saint Louis Missouri USA; ^2^ Department of Neurology University of Southern California Los Angeles California USA; ^3^ Department of Medicine University of Southern California Los Angeles California USA; ^4^ Department of Pathology & Laboratory Medicine University of California Irvine California USA; ^5^ Department of Radiology Brain Health Imaging Institute Weill Cornell Medicine New York New York USA; ^6^ Center for Genetic Epidemiology, Department of Population and Public Health Sciences, Keck School of Medicine University of Southern California Los Angeles California USA; ^7^ Alfred E. Mann School of Pharmacy and Pharmaceutical Sciences University of Southern California Los Angeles California USA; ^8^ Rush Alzheimer's Disease Center Rush University Chicago Illinois USA

**Keywords:** Alzheimer's disease, amyloid, amyloid‐related imaging abnormalities, apolipoprotein E, cerebral amyloid angiopathy, cytosolic phospholipase A2, neuroinflammation, phospholipase, *PLA2G4A*

## Abstract

Anti‐amyloid therapies for Alzheimer's disease (AD) modestly slow cognitive decline but carry significant risk of amyloid‐related imaging abnormalities (ARIAs), brain swelling, and hemorrhage, particularly in *apolipoprotein E ε4* carriers. Cerebral amyloid angiopathy (CAA) and vascular inflammation drive this vulnerability, highlighting the need for complementary strategies targeting upstream mechanisms of vascular injury. Cytosolic phospholipase A2 (cPLA2) regulates arachidonic acid and lysophosphatidylcholine‐derived lipid signaling at the intersection of amyloid burden, oxylipin dysregulation, blood–brain barrier disruption, and neurovascular inflammation. By depleting protective membrane plasmalogens while amplifying inflammatory lipid mediators, cPLA2 creates a state of vascular vulnerability predisposing to ARIAs. This Perspective article synthesizes evidence from human, preclinical, and translational studies positioning cPLA2 as an upstream driver of CAA‐related inflammation and vascular vulnerability in AD. We discuss biomarker and imaging approaches to assess cPLA2 activity in vivo and outline how targeting this pathway may enhance anti‐amyloid therapy safety by mitigating ARIA risk.

## Highlights

- Vascular inflammation and cerebral amyloid angiopathy contribute to Alzheimer's disease (AD) progression, particularly in *apolipoprotein E ε4* carriers.
- Cytosolic phospholipase A2 (cPLA2) regulates arachidonic acid–derived lipid pathways linked to neurovascular inflammation.
- Observational and experimental studies associate increased cPLA2 activation with amyloid‐related vascular pathology.
- The limited success of downstream anti‐inflammatory therapies supports reconsideration of upstream lipid regulators.
- cPLA2 inhibition is proposed as a potential adjunct strategy for targeting vascular inflammation in AD.

## 1 INTRODUCTION

Anti‐amyloid antibody therapies represent a therapeutic breakthrough in Alzheimer's disease (AD), but their clinical utility is limited by amyloid‐related imaging abnormalities (ARIAs)—brain swelling and hemorrhage that occur particularly in *apolipoprotein E* (*APOE*) *ε4* carriers, who face a 3‐ to 4‐fold higher risk than non‐carriers.^1^ The molecular mechanisms underlying this genotype‐specific vascular vulnerability remain poorly understood, limiting our ability to predict ARIA risk or develop protective interventions.

**FIGURE 1:**
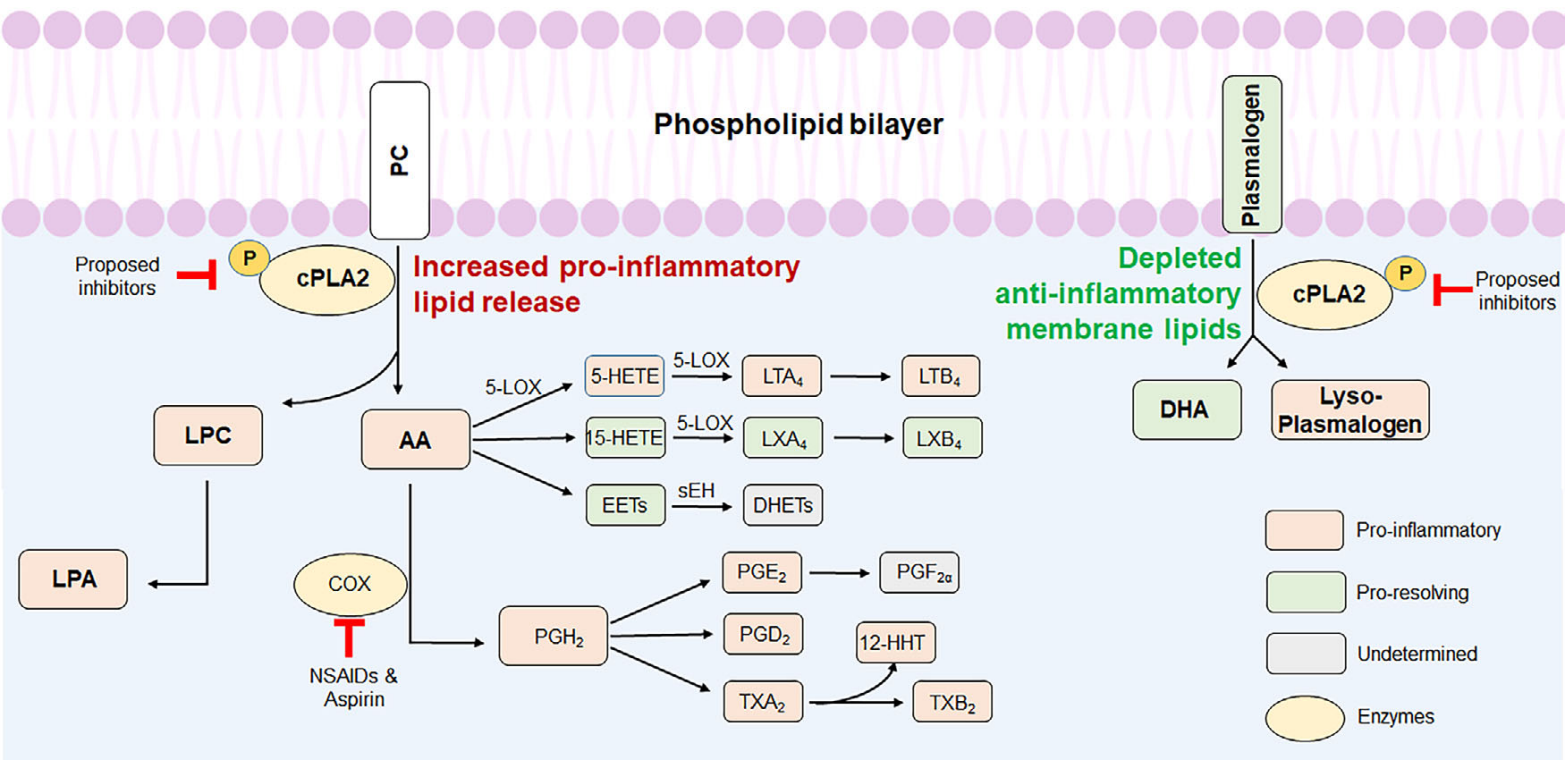
cPLA2 liberates PC to produce AA and the LPC pathway and depletes membrane plasmalogens, reducing DHA and releasing lyso‐plasmalogens. cPLA2 mediates the production of various oxylipins, including proinflammatory and proresolving lipids, through oxygenation of PUFAs. Orange squares denote inflammatory constituents, whereas green squares symbolize proresolving mediators. Gray squares correspond to analytes for which biological activity has not been determined yet.^3^ 5‐LOX, 5‐lipoxygenase; 12‐HHT, 12‐hydroxyheptadecatrienoic acid; AA, arachidonic acid; COX, cyclooxygenase; cPLA2, cytosolic phospholipase A2; EET, epoxyeicosatrienoic acid; DHA, docosahexaenoic acid; DHET, dihydroxyeicosatrienoic acid; HETE, hydroxyeicosatetraenoic acid; LPA, Lysophosphatidic acid; LPC, lysophosphatidylcholine; LT, leukotrienes; LX, lipoxin; NSAID, non‐steroidal anti‐inflammatory drug; PC, phosphocholine; PG, prostaglandin; PTGS, prostaglandin–endoperoxide synthase; PUFA, polyunsaturated fatty acid; TX, thromboxane.

We propose that cytosolic phospholipase A2 (cPLA2, encoded by *PLA2G4A*) is a key upstream driver linking *APOE* ε4, cerebral amyloid angiopathy (CAA), and vascular inflammation in AD. cPLA2 cleaves arachidonic acid (AA) and lysophosphatidylcholine (LPC) from membrane phospholipids (Figure 1).^2^, ^3^ These products generate diverse bioactive lipid mediators—oxylipins—including prostaglandins, leukotrienes, thromboxanes, and epoxy‐fatty acids that regulate vascular function, barrier integrity, immune cell trafficking, and inflammatory resolution. Critically, cPLA2 activity depletes protective membrane lipids, including docosahexaenoic acid (DHA)‐containing plasmalogens,^4^, ^5^ which are essential for membrane stability, lipid raft formation, antioxidant defense, and signaling.^6^, ^7^ We hypothesize that this depletion of protective lipids combined with increased inflammatory lipid production reduces membrane resilience, weakens blood–brain barrier (BBB) integrity, and impairs inflammation resolution, thereby increasing susceptibility to vasogenic edema (ARIA‐E) and hemorrhage (ARIA‐H). *APOE* *ε4* likely amplifies this pathway by elevating baseline vascular inflammation and oxidative stress while limiting lipid repletion, shifting the neurovascular unit toward a state of membrane lipid insufficiency that heightens BBB vulnerability and ARIA risk.

Unlike cyclooxygenase inhibitors that have failed in AD trials,^8^, ^9^, ^10^, ^11^ cPLA2 inhibition offers a distinct strategy: simultaneous suppression of multiple inflammatory pathways (lipoxygenase, cytochrome P450‐derived mediators) while preserving anti‐inflammatory membrane lipids. This Perspective article synthesizes evidence from human, preclinical, and translational studies to position cPLA2 as both a mechanistic driver of *APOE* *ε4*‐associated vascular vulnerability and a rational therapeutic target for CAA and ARIA prevention during anti‐amyloid immunotherapy.

## 2 VASCULAR COMPLICATIONS IN AD

Cerebrovascular pathology in AD encompasses chronic hypoperfusion, white matter hyperintensities, microbleeds, and microvascular degeneration.^12^, ^13^, ^14^ This Perspective focuses on vascular inflammation—an active, lipid‐mediated process distinct from passive ischemic injury—as a therapeutically tractable component of AD pathology. *APOE* *ε4* carriers demonstrate disproportionate vascular inflammation, manifesting as BBB breakdown, CAA‐related perivascular infiltrates, and elevated ARIA risk during immunotherapy.^1^, ^14^, ^15^ The molecular basis of this inflammatory vulnerability remains poorly defined. The following sections define BBB dysfunction, CAA‐related inflammation (CAA‐ri), and ARIA mechanisms, establishing the context for subsequent analysis of cPLA2's role in these inflammatory processes.

### 2.1 The BBB and AD pathogenesis

The BBB is a highly selective semipermeable interface formed by brain endothelial cells, pericytes, and astrocytic endfeet.^1^, ^15^ In AD, BBB dysfunction is increasingly recognized as a primary driver of pathology rather than a secondary consequence of neurodegeneration. This neurovascular vulnerability is particularly pronounced in *APOE ε4* carriers, in whom *APOE* *ε4* impairs amyloid beta (Aβ) clearance and exacerbates vascular inflammation.^1^ Additionally, *APOE ε4* triggers the matrix metalloproteinase‐9 (MMP9)‐cyclophilin A (CypA) pathway, leading to elevated MMP9, which degrades tight junction proteins, such as occludin and claudin‐5.^16^ This “leaky” phenotype is further exacerbated by the activation of cPLA2, inducing a proinflammatory lipid cascade.^17^ While pericyte loss contributes to diminished capillary perfusion and hypoxia, the *APOE* *ε4* effect primarily shifts the endothelium toward a fragile, proinflammatory state.^1^, ^18^ This pre‐existing vascular fragility potentially serves as the foundational substrate for ARIAs observed in anti‐amyloid immunotherapies. As monoclonal antibodies clear Aβ from the parenchyma and vessel walls, the resulting mechanical and inflammatory stress on a compromised BBB leads to ARIAs.^14^

### 2.2 CAA‐related inflammation

**FIGURE 2:**
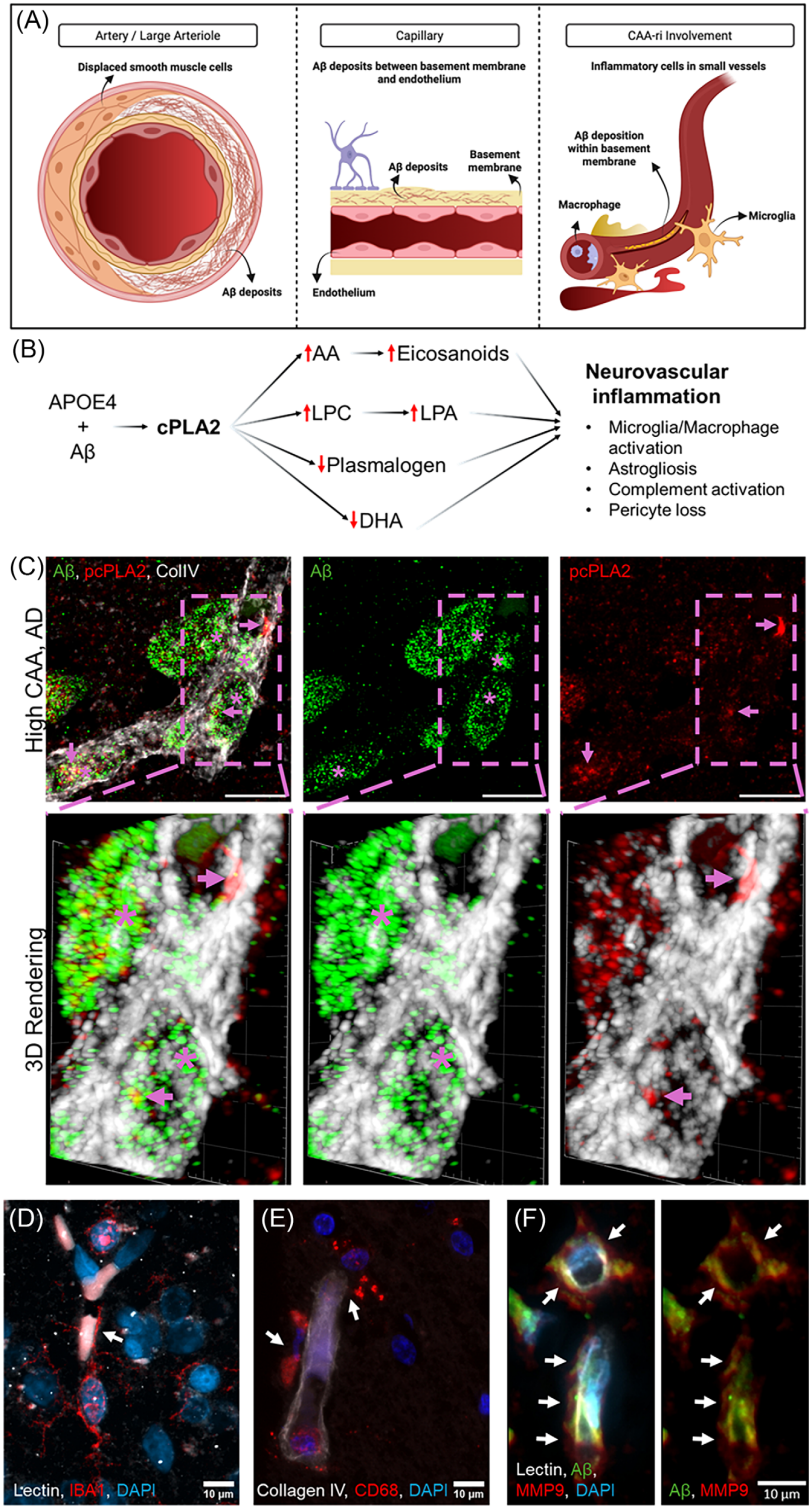
Aβ accumulation in the cerebral vessel walls is associated with cPLA2 activation and CAA‐ri. A, In CAA, amyloid deposits can accumulate in both large and small cerebral vessels displacing smooth muscle cells in the arteries and arterioles. In the capillaries, BBB is damaged and inflammation is triggered. B, We hypothesize that Aβ deposits in the cerebral vasculature along with *APOE* *ε4* activates cPLA2. cPLA2 releases inflammatory lipids and depletes anti‐inflammatory lipids, leading to neurovascular inflammation and BBB breakdown. C, High‐resolution images from high CAA, AD brain (CAA scores 3) showing Aβ deposition (*) and p‐cPLA2 (arrows) in and around the blood vessels (ColIV). Enlarged 3D rendering of the vessel, bottom panels, shows cPLA2 activity at the vicinity of the Aβ deposits (*) within (arrows) and surrounding the vessel. D, Microglia (IBA1) extend processes (arrow) to the endothelial cells (lectin). E, Pan‐macrophage marker, CD68, visualizes perivascular macrophages and microglia (arrows). F, MMP9 (arrows) was observed along the Aβ‐deposited cerebral vasculature (lectin). The right panel shows Aβ and MMP9 only. In all panels, DAPI marks the nuclei. Scale bars are 10 µm. Formalin‐fixed paraffin‐embedded sections from the midfrontal lobe were stained with the primary antibodies. AA, arachidonic acid; Aβ, amyloid beta; AD, Alzheimer's disease; *APOE*, apolipoprotein E; BBB, blood–brain barrier; CAA, cerebral amyloid angiopathy; CAA‐ri, cerebral amyloid angiopathy–related inflammation; cPLA2, cyclic phospholipase A2; DAPI, 4',6‐diamidino‐2‐phenylindole; IBA1, ionized calcium‐binding adapter molecule 1; LPA, Lysophosphatidic acid; LPC, lysophosphatidylcholine; MMP9, matrix metalloproteinase 9.

CAA is a small vessel disease (SVD), common in the aging brain and associated with cognitive decline. Independent of AD. It is characterized by the progressive accumulation and deposition of Aβ located in the cerebral vasculature within the cerebral cortex and leptomeninges (Figure 2A).^19^, ^20^ While neuropathological studies show that CAA is nearly ubiquitous among older adults with AD, particularly those who are *APOE* *ε4* carriers, CAA often remains subclinical or as an incidental finding on brain imaging.^21^, ^22^ Clinical presentation in the minority of individuals with symptomatic CAA, usually in the context of intracerebral hemorrhage complications of CAA, includes seizures, recurrent headaches, behavioral abnormalities, and cognitive decline.^23^ CAA is classified into two distinct subtypes depending on the cerebral vessel involvement. Type 1 CAA is predominantly associated with amyloid deposition in the basement membranes of cortical capillaries, as well as in the tunica media of cortical arteries, arterioles, veins, and leptomeningeal vessels. In contrast, in type 2 CAA, amyloid preferentially accumulates within the tunica media of leptomeningeal and larger cortical vessels, often with little to no deposits present in capillaries.^24^, ^25^ Amyloid accumulates in the tunica media and displaces smooth muscle cells. In general, Aβ40 peptide accumulates along the vessels in CAA, while Aβ42 is associated with amyloid plaques in AD. However, detailed immunohistological analysis revealed that Aβ42 also accumulates in cortical capillaries in type 1 CAA.^25^, ^26^, ^27^ Although type 2 CAA is more common in the aging brain, type 1 CAA is most often associated with AD pathology and perivascular inflammation, which is uncommon in type 2 CAA.

CAA‐ri is characterized by perivascular inflammation in response to Aβ deposition (Figure 2).^28^ CAA‐ri is associated with local BBB leakage and vessel rupture.^29^ Both *APOE* ε4 and Aβ oligomers drive the overactivation of cPLA2.^30^, ^31^ Eicosanoids, including PGE2 released by cPLA2, control MMP9 activation.^13^ MMP9–CypA pathway activation in pericytes degrades the BBB, resulting in low platelet‐derived growth factor beta (PDGFβ) tissue levels.^16^ In *APOE* *ε4* carriers, increased PDGFRβ, CypA, and MMP9 activity in cerebrospinal fluid (CSF) correlates with increased BBB permeability compared to *APOE* *ε3/ε3* individuals, independent of Aβ and tau pathology.^18^ Leaky BBB, increased MMP9 activity, impaired tight junctions, and reduced astrocyte end‐foot coverage of blood vessels are characteristic of the humanized *APOE ε4/ε4* mice compared to *APOE ε2/ε2* or *APOE ε3/ε3* mice.^15^ In addition, Aβ oligomers induce cPLA2 activation and neuroinflammation in mouse cortical neurons.^32^ Knocking out cPLA2 in a mouse AD model improves learning and memory, and decreases hyperactivity and premature death.^33^ Therefore, we hypothesize that CAA represents Aβ‐induced cPLA2 activation in cerebral vessel walls, resulting in CAA‐ri and BBB leakage, and that therapies reducing the activity of brain cPLA2, may also reduce cognitive impairment in aging (Figure 2). Because CAA is a major risk factor for ARIAs, diminishing cPLA2 activity may also lower ARIA risk in patients receiving anti‐amyloid therapies.^34^ In addition, Aβ can initiate the classical complement pathway.^35^ In the capillaries with CAA, Aβ and ApoE were present with the complement markers C1q and C3d. Membrane attack complex (MAC) induces cell lysis by creating holes in cell membranes.^29^ In CAA, MAC components C5b‐9 and C6 are associated with subcortical hemorrhage and cortical superficial siderosis.

## 3 CPLA2, OXYLIPINS, AND NEUROVASCULAR INFLAMMATION

### 3.1 Oxylipins and vascular inflammation

Oxylipins are bioactive lipid mediators derived from the oxygenation of polyunsaturated fatty acids (PUFAs), most notably AA (Figure 1).^3^ Beyond their generation, the release and turnover of oxylipins are regulated through a cascade of enzymatic steps. The process begins with phospholipase A2 (PLA2), which acts upstream by liberating AA and other PUFA substrates from membrane phospholipids. These free fatty acids are then metabolized by cyclooxygenases (COX‐1 and COX‐2), lipoxygenases (5‐LOX, 12‐LOX, and 15‐LOX), or cytochrome P450 monooxygenases, producing various oxylipins, such as prostaglandins (PGs), leukotrienes, hydroxy‐eicosatetraenoic acids (HETEs), and epoxyeicosatrienoic acids (EETs). Finally, downstream modulation of oxylipin activity, particularly epoxide‐containing species such as EETs, is controlled by soluble epoxide hydrolase (sEH), which hydrolyzes them into less active diols. The COX pathway leads to PGs and thromboxanes, which modulate vascular tone and inflammation. The LOX enzymes generate HETEs and leukotrienes, central to leukocyte recruitment and endothelial activation. The cytochrome P450 pathway produces EETs, which generally exert anti‐inflammatory and vasodilatory effects. Dysregulation of these oxylipins has been implicated in the pathogenesis of atherosclerotic cardiovascular disease, in which imbalances between proinflammatory (e.g., leukotrienes) and proresolving (e.g., lipoxins, EETs) oxylipins contribute to chronic vascular inflammation. Recently, specific oxylipin signatures have been correlated with inflammatory vascular phenotypes,^36^ and their release delays vascular recovery after endothelial injury.^37^

Recent studies have extended these findings to the brain, where oxylipins modulate neurovascular inflammation in diseases such as stroke and AD.^38^ AA‐derived oxylipins have been detected in CSF and brain tissue, showing altered profiles in neurodegenerative conditions. For example, higher levels of 12‐HETE and 15‐HETE were found in AD patients and were associated with microglial activation and cognitive impairment.^39^ In ischemic stroke, inhibition of sEH, which degrades vasoprotective EETs, reduced infarct size.^40^ Furthermore, elevated plasma levels of oxylipins from the linoleic acid pathway, including 12,13‐dihydroxyoctadecenoic acid (DiHOMEs), were associated with SVD and medial temporal lobe atrophy in the human brain, linking systemic oxylipin metabolism to cerebrovascular integrity.^41^ Together, these findings underscore the emerging role of oxylipins not only in systemic vascular inflammation but also in neurovascular diseases, offering new avenues for therapeutic targeting.

### 3.2 Plasmalogens and neuroinflammation

Plasmalogens are unique glycerophospholipids characterized by a vinyl‐ether bond at the sn‐1 position, a structure that confers specialized biophysical properties essential for maintaining neuronal membrane integrity and signaling pathways.^6^, ^7^ In AD, plasmalogen levels are significantly reduced in both the brain and circulation, a deficit increasingly recognized as a metabolic hallmark of the disease's progression.^7^ The biochemical consequences of plasmalogen depletion are multifaceted. Plasmalogens are critical components of lipid rafts, where they modulate the activity of secretases involved in amyloid precursor protein (APP) processing.^7^ Moreover, DHA‐containing plasmalogen depletion alters cell membrane lipid composition, where increased free cholesterol favors the amyloidogenic pathway.^7^, ^42^ Furthermore, the unique vinyl–ether bond allows plasmalogens to serve as sacrificial antioxidants, protecting neurons from oxidative stress, a primary driver of AD pathology.^7^, ^42^ In addition to reduced synthesis, plasmalogen degradation is important in the depletion of plasmalogens in AD.^6^, ^42^ Increased oxidative stress during AD progression can lead to plasmalogen degradation.^6^ Because DHA‐containing plasmalogens are regarded as sacrificial oxidants, their oxidation can protect the neurons until their depletion.^6^, ^7^ Among PLA2 enzymes, cPLA2 cleaves DHA‐containing plasmalogens and depletes them.^6^

### 3.3 cPLA2 as a master regulator of brain lipids

**FIGURE 3:**
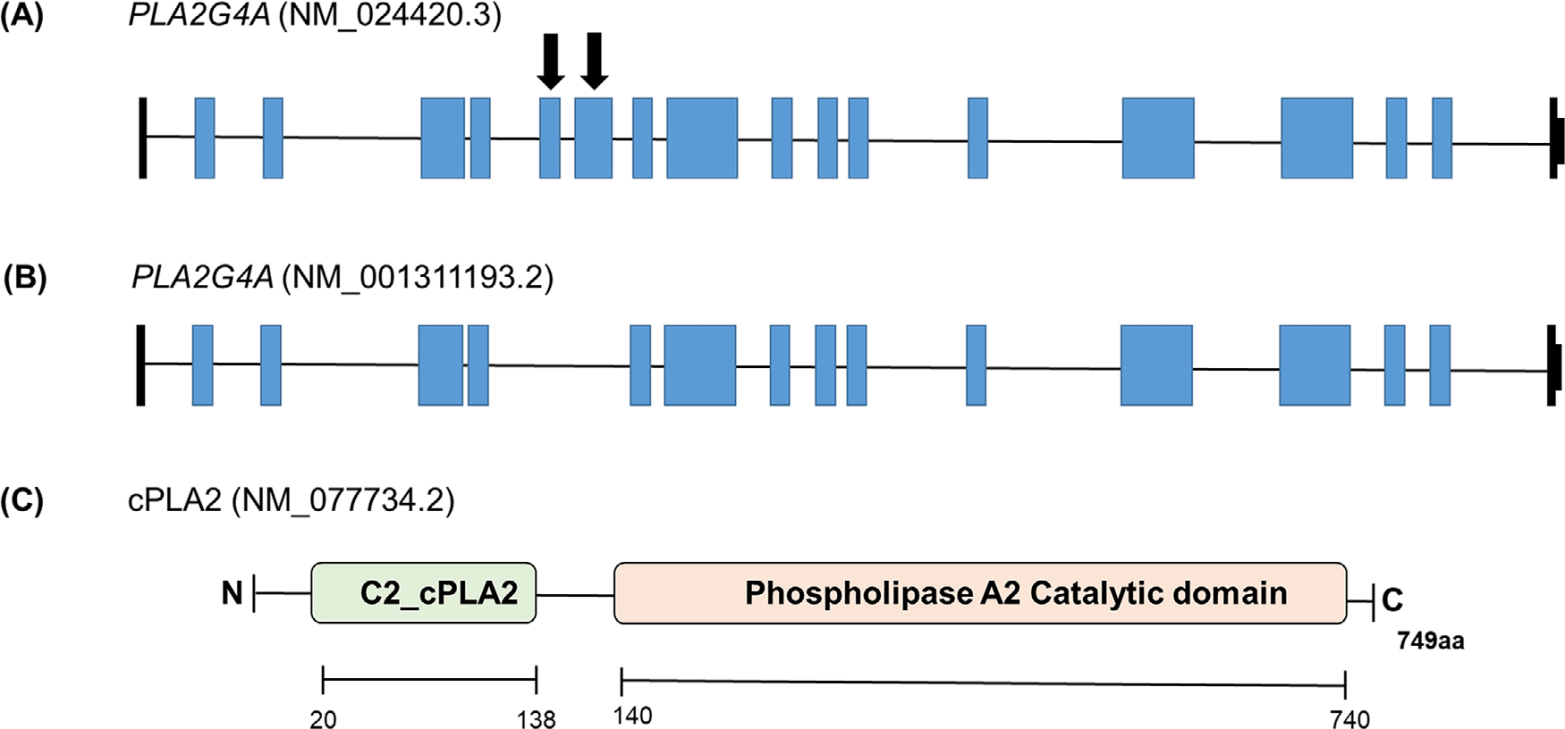
*PLA2G4A* isoforms and domains. A, *PLA2G4A* isoform (NM_024420.3) is the most common isoform consisting of 18 exons and encoding a protein with 749 amino acids. Blue‐colored boxes indicate coding exons, and black boxes denote non‐coding exons. Arrow indicates the sixth and seventh exons, which are missing in isoform NM_001311193.2. B, *PLA2G4A* isoform (NM_001311193.2) containing 16 exons. C, Schematic representation of cPLA2 protein (NP_000199.2) with two domains. C2_cPLA2 domain is involved in binding with calcium and phospholipase A2 catalytic domain has been reported to perform the function of calcium dependent lysophospholipase and phospholipase activities. These activities play a crucial role in the biosynthesis of lipid mediators and remodeling of lipids on cell membranes.^115^ cPLA2, cyclic phospholipase A2.

cPLA2, a 749 amino acid (85 kDa) protein is encoded by *PLA2G4A*. *PLA2G4A* (OMIM: 600522), also referred as *PLA2G4* (phospholipase A2 group IV), is located on chromosome 1q31.1 (Figure 3A and B).^2^ The cPLA2 protein consists of two domains, a calcium‐binding C2_cPLA2 domain and a phospholipase A2 catalytic domain (Figure 3C).^43^ *PLA2G4A* is highly expressed in different regions of the human brain (Figure S1 in supporting information),^44^ especially the mediodorsal nucleus of thalamus (MD), amygdala (AMY), and neocortex (NCX; Figure S2 in supporting information),^45^ whereas its subcellular localization includes the cytoplasm, Golgi apparatus, plasma membrane, and nuclear envelope.^46^ cPLA2 has a greater affinity to AA at the sn‐2 positions in phosphocholine (PC) and DHA at the sn‐2 positions of plasmalogens.^4^ Therefore, increased cPLA2 activity can augment the production of eicosanoids and LPC, while depleting membrane DHA and plasmalogens (Figure 1).

### 3.4 *PLA2G4A* conservation reveals its significant role in evolution

The Human Gene Mutation Database has reported only 12 pathogenic variants of *PLA2G4A*, including seven missense variants, three insertion/deletions, and two repeat variations (Table 1).^47^ However, the Leiden Open Variation Database reported only seven variants, of which only one variant *PLA2G4A* p.(Trp232CysfsTer24), was considered likely to be pathogenic.^58^ On the other hand, the ClinVar National Center for Biotechnology Information reported 200 germline variants in *PLA2G4A*, out of which two variants were classified as likely pathogenic and 24 variants were assessed as pathogenic.^59^ These findings indicate that *PLA2G4A* is associated with a cytosolic phospholipase‐A2 alpha deficiency and is associated with developmental disorders. Overall, the above studies indicate that the sequence of *PLA2G4A* is highly conserved and mutations in this gene are penetrant most of the time. *PLA2G4A* shows low tolerance for loss‐of‐function variants yet high conservation across species, underscoring its indispensable role in development; however, this does not preclude increased activation as a precursor for chronic inflammatory diseases of aging, including AD.

**TABLE 1:** Previous *PLA2G4A* variants and their associated phenotypes with comorbidities.

Chromosomal position	Variant	Transcript *PLA2G4A*	Variant type	Phenotype	Comorbidities related to dementia	Reference
Chr1:186894164 T > C	c.331 T > C; p.Ser111Pro	NM_024420.3	Missense/nonsense	Prostanoid biosynthesis deficiency	Not reported	^48^, ^49^, ^50^
Chr1:186932824 G > T	c.620 G > T; p.Gly207Val	NM_024420.3	Missense/nonsense	Developmental disorder	Not reported	^51^, ^52^
Chr1:186956219 G > A	c.145G > A; p.Arg485His	NM_024420.3	Missense/nonsense	Prostanoid biosynthesis deficiency	Not reported	^48^, ^49^, ^50^
Chr1:186965438 C > T	c.1609C > T; p.Pro537Ser	NM_024420.3	Missense/nonsense	Developmental disorder	Not reported	^51^, ^52^
Chr1:186965552 G > C	c.1723G > C; p.Asp575His	NM_024420.3	Missense/nonsense	Gastroduodenal ulcers	Not reported	^53^
Chr1:186977668 C > T	c.1840C > T; p.Arg614Trp	NM_024420.3	Missense/nonsense	Developmental and epileptic encephalopathy	Not reported	^54^
Chr1:186977780 G > A	c.1952A > G; p.Lys651Arg	NM_024420.3	Missense/nonsense	Increased enzyme activity	Not reported	^49^
Chr1:186932809 TG > T	c.607delG; p.Val203Trpfs*6	NM_024420.3	Small deletion	Developmental disorder	Not reported	^55^
Chr1:186956142 TAATC > T	c.1380_1383delTCAA; p.Asn460Lysfs*8	NM_024420.3	Small deletion	Developmental disorder	Not reported	^51^, ^52^
Chr1:186979472 TGTAA > T	c.2118+4_2118+7delAGTA	NM_024420.3	Small deletion	Cryptogenic multifocal ulcerating stenosing enteritis	Not reported	^56^
NA	c.−180T > C	NM_024420.3	Repeat variation	Associated with severe asthma	Not reported	^57^
NA	c.−185A > C	NM_024420.3	Repeat variation	Associated with severe asthma	Not reported	^57^

Abbreviation: NA, not available.

### 3.5 cPLA2 activation in neuroinflammation and AD

Elevated cPLA2 levels are associated with increased production of proinflammatory cytokines and neuroinflammation.^60^ In cyclin‐dependent kinase 5 (Cdk5)–induced neuroinflammation, increased cPLA2 expression enhances LPC secretion, which ultimately leads to neurodegeneration in murine models. cPLA2 knock‐down rescues neuroinflammation in these mice.^60^ Knocking out *PLA2G4A* from microglia in murine models reduces inflammatory response induced by lipopolysaccharides via reactive oxygen species (ROS)/nitric oxide (NO) signaling through the lipoxygenase pathway.^61^ cPLA2 activation disrupts fatty acid metabolism, the nitrosylation pathway, and neuronal excitation, leading to neurodegeneration.^62^ In AD brains, phosphorylated (activated form of) cPLA2 levels are significantly increased compared to healthy controls.^63^ cPLA2 activation is a critical driver of neuroinflammation and oxidative stress in AD, particularly in individuals with the *APOE* *ε4* genotype.^31^ In primary astrocytes and brains of *APOE ε4/ε4* mice, cPLA2 phosphorylation and activity, leukotriene levels, and highly phosphorylated p38 MAPK are increased compared to *APOE ε3/ε3* mice.^31^ These findings are confirmed in *post mortem* human tissues. cPLA2 expression is elevated in astrocytes surrounding Aβ plaques in AD human brains, compared to healthy controls.^64^ In addition, Aβ oligomers induce cPLA2 activation and neuroinflammation in mouse cortical neurons.^32^ In vitro, soluble Aβ oligomer‐induced neurotoxicity is mediated by cPLA2 activation and can be alleviated by cPLA2 inhibition.^30^, ^65^ In two AD mouse models that overexpress Aβ, cPLA2 deficiency ameliorates symptoms of cognitive deficits and improves learning and memory.^33^, ^66^ Esterification of cholesterols exacerbates Aβ‐induced signaling, which is crucial for cPLA2 activation. Activation of this pathway is associated with synaptic degeneration and the development of cognitive impairment, thereby intensifying the onset and progression of AD.^67^, ^68^, ^69^

## 4 THERAPEUTIC IMPLICATIONS

### 4.1 Clinical evidence for targeting AA‐derived oxylipins in neuroinflammation

It is estimated that 20% of all new drugs in the development pipeline for AD are focused on various aspects of neuroinflammation.^70^ Existing drugs that target brain inflammation based on the AA pathway in neurodevelopmental disorders (NDDs) include acetylsalicylic acid (ASA) and COX inhibitors.^8^ Until now, these drugs were not effective against AD. Below, we discuss findings from ASA and COX inhibitor trials and offer insights for their lack of effectiveness.

#### 4.1.1 ASA

ASA (aspirin) was first synthesized in 1897 as an acetylated derivative of salicylate and was used for its anti‐inflammatory and antiplatelet properties.^71^ ASA functions as a non‐selective COX inhibitor, inhibiting the biosynthesis of PGs and impeding proinflammatory cytokine activity (Figure 1).^71^ Previously, ASA was found to reduce the formation of neurofibrillary tangles and tau phosphorylation in neural cells in vitro.^72^ In another study, ASA was found to lower NO production and protect neurons from oxidative stress in vitro.^73^ Recently, a transgenic AD mouse model with amyloid pathology (APP/PS1) revealed that ASA treatment partially rescued the expression of CDK1/2/4, p18/21, and cyclin A2/B1/D3/E1 and decreased neuronal apoptosis.^74^ Further, in the same murine model, ASA induced Aβ degradation, reduced interleukin 6 and tumor necrosis factor alpha (TNF‐α) release, and enhanced learning and memory.^74^ Despite these preclinical observations, clinical trial data supporting ASA's use against neuroinflammation in AD is lacking. The Aspirin in Reducing Events in the Elderly trial was a randomized, double‐blind, placebo‐controlled study of 19,114 healthy older adults (mean age ≥ 70) without cardiovascular disease or dementia at baseline.^10^ Participants received a low‐dose aspirin (100 mg) or placebo daily and were followed for a median of 4.7 years.^10^ In a prespecified secondary analysis focused on cognitive outcomes, aspirin showed no benefit in preventing dementia, mild cognitive impairment, or cognitive decline.^10^ However, the trial was not conducted in a population with neuroinflammation nor was a reduction in neuroinflammation analyzed after ASA treatment. This highlights the difficulty of targeting a diverse elderly population. The absence of specific neuroinflammation biomarkers at baseline, illustrates the challenge in evaluating whether aspirin effectively reduces neuroinflammation or not.

#### 4.1.2 COX inhibitors

COX‐2 is integral to the biosynthetic pathway of PGs and serves as an essential mediator in the modulation of neuroinflammation, nociception, and vascular homeostasis.^75^ It is targeted by many other non‐steroidal anti‐inflammatory drugs (NSAIDs; Figure 1). Multiple studies have revealed the association of COX‐2 with tau, Aβ, acetylcholine, and many kinases associated with AD such as glycogen synthase kinase 3β and cyclin‐dependent kinase 5.^76^ COX‐2 is expressed in cortical and hippocampal neurons and is linked to long‐term synaptic plasticity, neuroinflammation, brain injury, and neurological disorders such as AD, multiple sclerosis (MS), Parkinson's disease (PD), and epilepsy.^76^ COX‐2 inhibition has long been discussed as a potential therapeutic target for the management of AD.^76^ Although numerous studies have shown that COX‐2 plays a critical role in reducing long‐term potentiation, which is essential for memory formation, the effectiveness of NSAIDs in preventing dementia and AD remains unclear.^76^ Some studies have even suggested that paracetamol (acetaminophen), a common NSAID, might increase the risk of developing these conditions rather than preventing them.^77^, ^78^, ^79^ Administration of COX‐2 inhibitors has also been reported to have side effects on cardiovascular health, memory, and cognitive functions,^80^, ^81^ pinpointing the need to develop other therapeutic targets. Investigations have also implicated COX‐1, one of the isoforms with beneficial effects on mitigating neuroinflammation and neurodegenerative conditions.^82^ Recently, a COX‐1‐ and COX2‐inhibitor “ketorolac”, which is a non‐steroidal, non‐selective, Food and Drug Administration‐approved anti‐inflammatory drug, was identified as a repurposed drug for AD.^83^ Single nucleus RNA sequencing data from 0.7 million AD patients and controls was analyzed and *SYK*, *CTSB*, and *INPP5D* genes were identified as molecular drivers responsible for the transition from disease associated microglia to neuroinflammation‐like microglia. Ketorolac was predicted to target microglial inflammation using network‐based approach.^83^ However, in randomized trials, COX inhibitors were found to have limited anti‐neuroinflammatory effects.^84^ COX inhibitors uniformly failed to demonstrate benefit—and in some cases suggested harm—despite encouraging observational data. The Alzheimer's Disease Anti‐inflammatory Prevention Trial randomized 2528 cognitively normal older adults to naproxen (220 mg BID), celecoxib (200 mg BID), or placebo.^9^ Treatment was suspended after ≈ 2 years when safety concerns emerged from the trials due to increased cardiovascular risk.^9^ Interim analyses revealed no reduction in incident AD or cognitive decline; in fact, both active‐treatment arms trended toward higher AD rates and showed worsened performance on global cognitive scores compared to placebo.^11^, ^85^ NSAIDs such as ASA and selective COX‐2 inhibitors target a single downstream arm of the AA cascade, the COX‐mediated PG production, yet they leave intact parallel proinflammatory pathways generated by lipoxygenases and cytochrome P450s.^31^ To mitigate a compensatory unintended activation of this pathway, inhibiting cPLA2 prevents the formation of PGs, leukotrienes, thromboxanes, and other eicosanoids.^86^ Theoretically, cPLA2 inhibitors promise a more comprehensive dampening of neuroinflammation, with the potential to avoid compensatory activation of bypass pathways and achieve greater therapeutic efficacy. Next, we review the literature and challenges for targeting cPLA2 for NDDs such as AD.

### 4.2 Targeting cPLA2 activity to reduce neuroinflammation

cPLA2 activity has been targeted in multiple NDDs other than AD, such as PD, PD dementia, MS, and traumatic brain injury (TBI).^87^ MS is a neuro‐inflammatory disease, and the AA pathway is activated in the brains of patients with MS.^88^ Indeed, in MS animal models, cPLA2 inhibition or genetic knock‐out reduces disease severity and protects against MS, as shown in multiple studies.^89^, ^90^ In addition, cPLA2 plays a significant role in TBI‐induced lysosomal membrane permeabilization (LMP). In rodents, LMP manifests in neurons subsequent to TBI.^91^ LMP resulted in compromised macroautophagy and neuronal apoptosis. The comparison of lysosomal membrane lipid profiles between TBI and sham animals revealed that the activation of cPLA2‐induced LMP leading to autophagy inhibition in primary neurons and human H4 neuroglioma cell line.^91^ Pharmacological suppression of cPLA2 in vivo resulted in a reduction of TBI‐induced LMP, alongside a subsequent attenuation of autophagic dysfunction and neuronal cell death, correlating with enhanced neurological recovery outcomes.^91^ The inhibition of cPLA2 in vitro also restricted Aβ‐induced LMP and impeded autophagy processes, revealing that cPLA2‐mediated damage to lysosomal membranes plays a significant role in neuronal cell mortality in neurodegenerative pathologies, including AD, and inhibiting cPLA2 expression could reduce neuronal mortality.^91^ Among the small molecules that target cPLA2, arachidonyl trifluoromethyl ketone (7.5 mg/kg) was orally administered to rats for the treatment of neuroinflammation‐associated morbidities in lumbar spinal cord stenosis (LSS).^92^ After treatment, the LSS rats showed reduced pain threshold, improved functional deficits, and locomotion. Lipid profiles of treated animals versus control groups revealed a reduction in the levels of proinflammatory lipid mediators, such as free fatty acids, LPC, PG E2, and leukotrienes B4.^92^ In addition, few studies have revealed that long‐term inhibition of cPLA2 is favorable in the recovery from NDDs; however, caution would be needed to select different types of inhibitors and their concentrations.^93^

### 4.3 cPLA2 reduction protects BBB function in vascular brain disorders

cPLA2 plays a critical role in microglial activation in murine models suffering from cerebral infarction.^94^ cPLA2 activity also increases in rats suffering from cerebral ischemia. Elevated cPLA2 leads to increased TNF‐α and NO production and activation of microglia, which ultimately leads to increased neuronal apoptosis.^95^ In animal stroke models, the genetic loss of cPLA2 dramatically decreases poststroke neuroinflammation and postischemic reperfusion injury.^96^ In rat models of epilepsy, cPLA2 interacts with glutamate in brain capillaries and enhances the expression of P‐glycoprotein (P‐gp) and breast cancer resistance protein, a process that is dependent on the cPLA2–AA–Cox‐2 pathway and ultimately disturbs BBB.^97^ Knockout or inhibition of cPLA2 in the capillaries reverses this phenotype and protects the BBB. In primary murine cerebral endothelial cells and astrocytes, Aβ42 activates the MAPK/ERK pathway, leading to the phosphorylation of cPLA2.^98^ In endothelial cells, ROS production is increased as well. In turn, ROS and cPLA2 activity are believed to impair the BBB. Endothelial cPLA2 is also involved in the BBB maturation through pericyte‐secreted factors.^99^ These factors activate PKC/MAPK/ERK signaling pathway, which in turn phosphorylates and activates cPLA2 in pericyte cocultures.^99^ Pericytic MMP9–CypA pathway activation degrades the BBB, resulting in low PDGFRβ levels.^16^ Increased sPDGFRβ, CypA, and MMP9 activity correlates with increased BBB permeability in *APOE* *ε4* carriers compared to *APOE* *ε3/ε3* individuals, independent of Aβ and tau pathology.^18^ Leaky BBB, increased MMP9 activity, impaired tight junctions, and reduced astrocyte end‐foot coverage of blood vessels are observed in the humanized *APOE ε4/ε4* mice.^15^ Atorvastatin treatment dampened neuroinflammation, oxidative stress, and BBB damage through downregulating 12/15‐LOX, p38MAPK, and cPLA2 activity. In an in vitro BBB model, hypoxia‐induced inflammation in human brain microvascular endothelial cells is mitigated by decreasing cPLA2 activity via the nuclear factor kappa beta pathway.^100^ In conclusion, targeting cPLA2 activity could be a promising therapeutic strategy for protecting against neuroinflammation and maintaining BBB integrity in conditions such as cerebral infarction, ischemia, epilepsy, and AD.

### 4.4 The case for cPLA2 activation in CAA‐ri

CAA‐ri reflects a vascular inflammatory response to Aβ deposition within cerebral vessels and represents a key intersection between cerebrovascular pathology and AD. We propose that cPLA2 activation is a central upstream driver of this process. In *APOE* *ε4* carriers, vascular Aβ accumulation induces endothelial and perivascular stress signaling that promotes calcium‐dependent phosphorylation of cPLA2. Activated cPLA2 liberates AA and LPC, triggering local production of proinflammatory oxylipins that amplify vascular inflammation, activate MMP9, and disrupt BBB integrity. Complement activation by vascular Aβ may further reinforce cPLA2 signaling, creating a feed‐forward cycle of vascular injury. This lipid‐driven inflammatory cascade provides a mechanistic framework linking CAA‐ri to BBB leakage, microhemorrhages, and heightened susceptibility to ARIAs during anti‐amyloid therapy. One of these mechanisms can include C5b‐9 triggering cPLA2, which in turn can lead to cellular damage in the glomerular epithelial cells.^101^, ^102^, ^103^ This activation depends on cPLA2 phosphorylation and membrane translocation.^101^ Activation of the complement system by Aβ and APOE possibly leads to MAC formation and cPLA2 activation in these vessels. AA and LPC released by cPLA2 activation may further exacerbate vessel damage by starting a chain of inflammation leading to CAA‐ri and hemorrhages.

## 5 BIOMARKER DEVELOPMENT AND PATIENT STRATIFICATION

Effective cPLA2‐targeted therapy requires biomarkers to identify patients with elevated inflammatory activity and monitor target engagement. CSF and plasma oxylipin profiling offers direct readouts of cPLA2 activity. In AD patients, elevated 12‐HETE, 15‐HETE, and altered PG ratios correlate with disease severity and *APOE* *ε4* genotype.^39^, ^41^ These signatures may predict ARIA susceptibility in patients receiving anti‐amyloid therapy, enabling risk stratification and personalized dosing strategies.

Neuroimaging provides complementary non‐invasive assessment. [^1^ ^1^C]‐AA positron emission tomography (PET) demonstrates increased AA incorporation in the AD cortex, particularly in regions with dense neuritic plaques and activated microglia.^104^, ^105^ We developed [^1^ ^8^F]‐labeled AA and DHA tracers with improved half‐life for clinical translation.^17^, ^106^, ^107^ Preliminary studies in *APOE ε4* knock‐in mice show elevated tracer uptake consistent with heightened cPLA2 activity, positioning [^1^ ^8^F]‐AA and DHA PET as a tool for detecting early inflammation, monitoring disease progression, and evaluating cPLA2 inhibitor efficacy.

Target engagement biomarkers from peripheral blood are also feasible. Plasma AA levels, PGE2, and leukotriene B4 can confirm cPLA2 inhibition.^86^, ^108^, ^109^ This approach has precedent: a Phase 2 trial of the 5‐lipoxygenase inhibitor VIA‐2291 successfully used LTB4 as a pharmacodynamic marker.^110^ Integrating genotype (*APOE* *ε4*), biomarkers (CSF oxylipins, PET imaging), and clinical phenotype (ARIA history) will enable precision application of cPLA2 inhibitors to high‐risk populations.

## 6 CHALLENGES AND CONTROVERSIES

### 6.1 Clinical development considerations

Translating cPLA2 inhibition to the clinic presents several surmountable challenges. First, achieving selectivity for cPLA2 over other phospholipase A2 isoforms is critical to minimize off‐target effects. Early inhibitors suffered from poor selectivity and rapid metabolic clearance. However, structure‐based design and recent medicinal chemistry advances have yielded compounds with > 100‐fold selectivity for cPLA2.^111^ The challenge lies in balancing lipophilicity for BBB penetration against metabolic stability and toxicity.

Safety concerns center on immunosuppression from complete cPLA2 inhibition with immune dysfunction or bleeding disorders observed with loss‐of‐function mutations (Table 1). However, partial inhibition may preserve immune function while reducing pathological inflammation. The cPLA2 inhibitor AK106‐001616, tested in rheumatoid arthritis at doses up to 600 mg for 28 days, demonstrated acceptable tolerability with manageable side effects comparable to high‐dose naproxen.^112^, ^113^ This establishes proof of concept for partial cPLA2 inhibition in humans, though central nervous system (CNS) penetration was not assessed in that trial. BBB penetration represents a critical requirement. Computational modeling and prodrug strategies may optimize brain delivery. In addition, development of pharmacodynamic biomarkers (plasma AA, CSF oxylipins, PET imaging) will enable dose optimization in early‐phase trials. The convergence of selective inhibitors, biomarker‐guided dosing, and high‐risk patient enrichment (*APOE* *ε4* carriers with MRI evidence of CAA or receiving anti‐amyloid therapy) creates a viable path to clinical proof‐of‐concept testing.

## 7 FUTURE DIRECTIONS

Translating cPLA2 biology into therapeutic application requires advances in three areas. First, brain‐penetrant inhibitors with selectivity for vascular cPLA2 over peripheral immune function are needed. Structure‐guided approaches targeting the C2 domain may yield allosteric modulators suitable for chronic CNS use. Preclinical studies in *APOE* *ε4* transgenic models should prioritize cerebrovascular endpoints, such as barrier permeability, microhemorrhage burden, and plasmalogen preservation, over traditional cognitive metrics. Second, biomarkers for target engagement must be validated. While plasma oxylipin profiling (PGE2, leukotriene B4) provides pharmacodynamic evidence, CSF plasmalogen levels and imaging markers of BBB integrity offer more direct readouts of therapeutic effect in the brain. PET tracers targeting activated cPLA2 could enable in vivo visualization of enzyme activity in CAA‐affected vessels. Third, clinical integration with anti‐amyloid immunotherapy represents the most immediate opportunity. Adaptive trials in *APOE* *ε4* carriers receiving lecanemab or donanemab could test whether cPLA2 inhibition reduces ARIA incidence, enabling safer dosing and broader treatment access. Early intervention in presymptomatic *APOE* *ε4* carriers with elevated amyloid burden may prevent both vascular injury and future ARIA risk. Some studies have also proposed two inflammatory stages of AD: a preclinical (early) stage, which appears to be amenable to anti‐inflammatory therapy, and a clinical (late) stage, which is less amenable.^114^ Targeting the preclinical stage with precise anti‐inflammatory agents could lead to a breakthrough in slowing down vascular complications of AD.^85^

## 8 CONCLUSION

cPLA2 represents a convergence point for three pathological processes driving ARIA risk in AD: amyloid‐triggered vascular inflammation, such as CAA‐ri, *APOE* *ε4*‐mediated lipid dysregulation, and BBB breakdown. By depleting protective membrane plasmalogens while amplifying proinflammatory oxylipin production, cPLA2 activity creates a state of vascular vulnerability that predisposes to edema and hemorrhage during amyloid clearance. This mechanistic framework positions cPLA2 inhibition as a rational strategy for CAA‐ri and to enhance the safety and tolerability of anti‐amyloid immunotherapy, particularly in *APOE* *ε4* carriers who face the highest ARIA burden. While challenges remain—including inhibitor selectivity, brain penetration, and preservation of beneficial lipid signaling—evidence from human *post mortem* lipidomic studies, preclinical CAA models, and the enzyme's central role in membrane remodeling provides a compelling rationale for clinical translation.

## CONFLICT OF INTEREST STATEMENT

The authors declare that the research was conducted in the absence of any commercial or financial relationship that could be construed as a potential conflict of interest. Author disclosures are available in the supporting information.

## Supporting information



Supporting information

Supporting information
