# Supplementary material for: A perspective: PLA2G4A as drug target for vascular inflammation in Alzheimer's disease
Source: Alzheimers Dement. 2026 Apr 8;22(4):e71320. doi: 10.1002/alz.71320 (PMC13060764; doi:10.1002/alz.71320)
Supplement: Supplementary file 1 — Supporting information [file ALZ-22-e71320-s001.pdf]

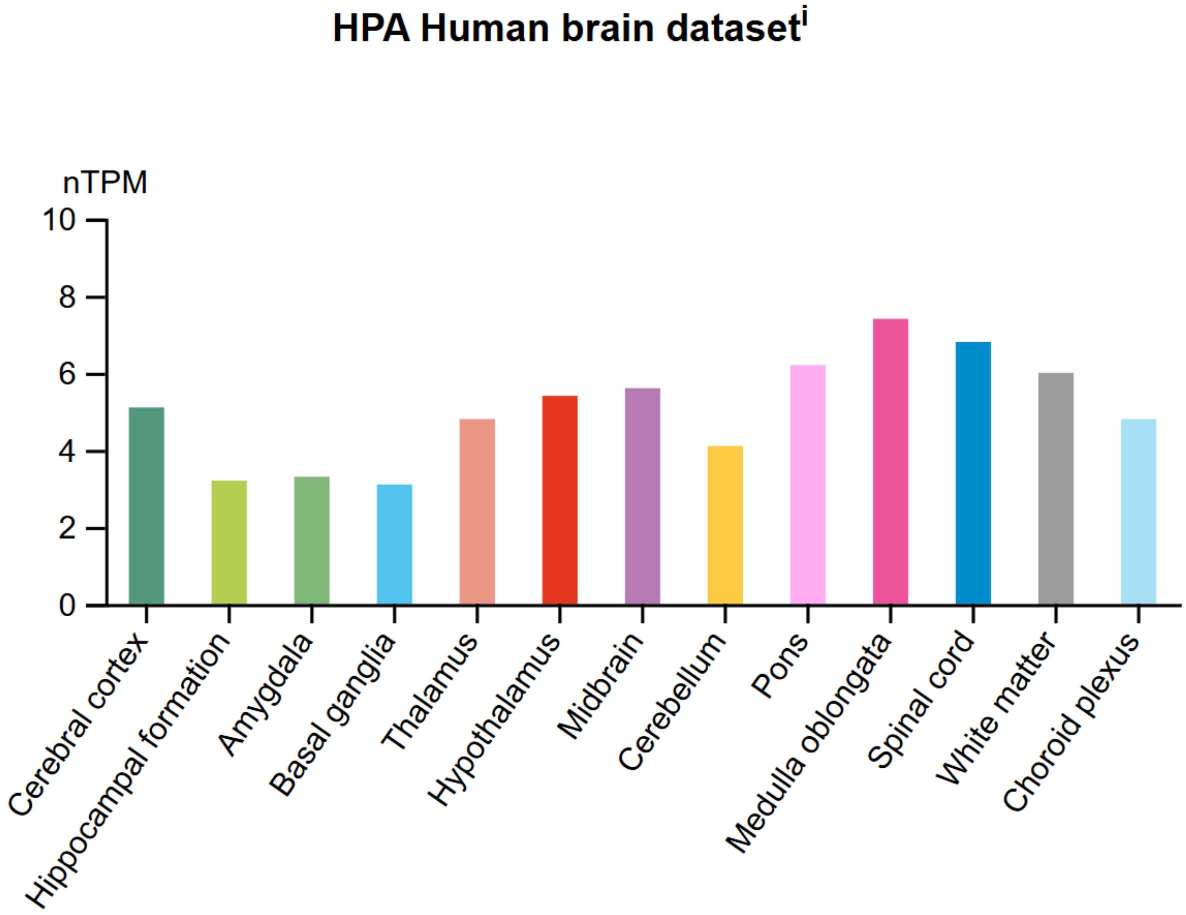

Supplementary Figure 1: The HPA Human brain dataset (https://www.proteinatlas.org/) provided the normalized expression levels of *PLA2G4A* RNA in transcripts per million (nTPM) for 13 regions of the human brain. Medulla oblongata exhibited the most prominent expression (7.4 nTPM) of *PLA2G4A*. The individual expression in each sub-region has been shown by the height of the each bar.

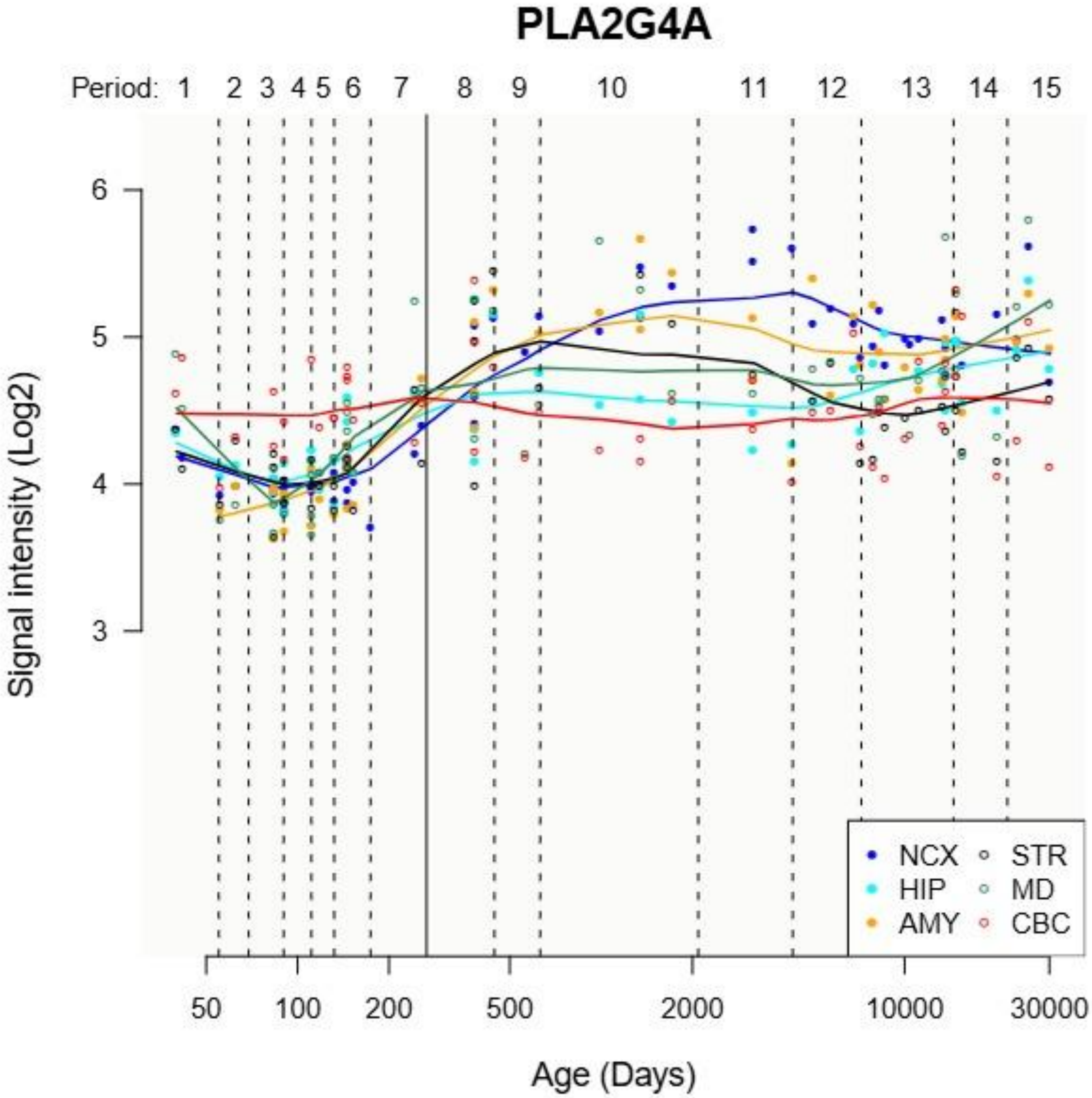

Supplementary Figure 2: Human brain transcriptome analysis reveals dynamic gene expression of *PLA2G4A* during development and adulthood in different regions of the brain (https://hbatlas.org/pages/hbtd). MD, mediodorsal nucleus of the thalamus, HIP, hippocampus, CBC, cerebellar cortex, STR, striatum, AMY, amygdale, NCX, 11 other areas of the neocortex. *PLA2G4A* has the highest expression in the mediodorsal nucleus of the thalamus (MD), amygdala (AMY), and neocortex (NCX) regions of the human brain.
